# Computed Tomography-Derived Psoas Muscle Index as a Diagnostic Predictor of Early Complications Following Endovascular Aortic Repair: A Retrospective Cohort Study from Two European Centers

**DOI:** 10.3390/jcm14155333

**Published:** 2025-07-28

**Authors:** Joanna Halman, Jan-Willem Elshof, Ksawery Bieniaszewski, Leszek Bieniaszewski, Natalia Zielińska, Adam Wójcikiewicz, Mateusz Dźwil, Łukasz Znaniecki, Radosław Targoński

**Affiliations:** 1 Department of Vascular Surgery, University Clinical Center and Faculty of Medicine, Medical University of Gdańsk, 80-214 Gdańsk, Poland; 2 Department of Vascular Surgery, VieCuri Medisch Centrum, 5912 BL Venlo, The Netherlands; 3 Department of Surgical Oncology Transplant Surgery, and General Surgery, Faculty of Medicine, Medical University of Gdańsk, 80-214 Gdańsk, Poland; 4 Clinical Physiology Unit, Medical Simulation Center, Medical University of Gdańsk, 80-204 Gdańsk, Poland; 5 Student Scientific Circle of Neurotraumatology, Medical University of Gdańsk, 80-210 Gdańsk, Poland; 6 Cardiology Clinic, University Clinical Center, 80-214 Gdańsk, Poland; radoslaw.targonski@gumed.edu.pl

**Keywords:** Psoas Muscle Index, Imagine Biomerkers, Sarcopenia

## Abstract

**Background/Objective:** Sarcopenia is a predictor of poor surgical outcomes in older adults. The Psoas Muscle Index (PMI), calculated from routine preoperative CT scans, has been proposed as an imaging-based marker of physiological reserve, but its diagnostic utility in vascular surgery remains unclear. We aimed to assess the predictive value of PMI for early complications following elective abdominal aortic aneurysm (AAA) repair in two European centers. **Methods:** We retrospectively analyzed 245 patients who underwent open or endovascular AAA repair between 2018 and 2022 in Poland and The Netherlands. PMI was measured at the level of third lumbar vertebrae (L3) level, normalized to height, and stratified into center-specific tertiles. Early complications were compared across tertiles, procedures, and centers. Multivariate logistic regression was used to adjust for age, comorbidities, and procedure type. **Results:** Low PMI was significantly associated with early complications in EVAR patients at the Polish center (*p* = 0.004). No associations were found in open repair or at the Dutch center. Mean PMI values did not differ significantly between centers. **Conclusions:** PMI may serve as a context-dependent imaging biomarker for early risk stratification following AAA repair, particularly in endovascular cases. Its predictive value is influenced by institutional and procedural factors, highlighting the need for prospective validation and standardization before clinical adoption.

## 1. Introduction

Patients undergoing vascular surgery are generally elderly and often have multiple comorbidities. A significant number of individuals experience a decline in skeletal muscle mass, strength, and quality, which is associated with poorer surgical outcomes. So far, routine sarcopenia assessment has not been included in preoperative evaluation because it requires specialized equipment or qualified personnel, who are not always available in vascular settings or centers. CT-derived measures, such as psoas muscle area (PMA) and psoas muscle index (PMI), have emerged as potential predictors of prognosis that are easy to assess on preoperative CT scans, can be performed by surgeons themselves, and add value to preoperative risk assessment by informing clinicians about the need to implement nutritional support, which can then improve outcomes, enhance wound healing, and increase survival [1,2].

Abdominal aortic aneurysm repair is usually an elective, preventive procedure aimed at decreasing the risk of rupture. The main challenge is not only determining which patients are most likely to benefit from the intervention but also choosing the best timing of the procedure. Notably, the elective nature of AAA repair provides time for preoperative optimization. In this setting, the PMI can be a valuable addition to existing risk assessment tools, helping to identify patients who might benefit from less invasive options such as EVAR, require specific prehabilitation strategies (e.g., nutritional support), or are too high-risk for surgery.

As vascular surgery increasingly adopts personalized, risk-based strategies, imaging biomarkers become valuable tools for evaluating physiological reserves. Despite its potential, the prognostic value of PMI in AAA repair remains uncertain. Most previous studies have been limited to single-center cohorts with varied designs and population-specific cut-off values, reducing their external validity and comparability. Additionally, a few have investigated whether the predictive value of PMI varies by surgical approach (open aortic repair (OAR) versus endovascular aneurysm repair (EVAR)) or across healthcare systems with different resources and perioperative protocols [2,3,4,5,6].

This study aims to evaluate the clinical utility of preoperative PMI in predicting early postoperative complications following AAA repair. By analyzing data from two distinct European vascular centers with differing patient populations, surgical strategies, and healthcare contexts, we sought to determine whether PMI is a robust and generalizable prognostic marker or whether its utility is context-dependent.

## 2. Materials and Methods

We conducted a multicenter retrospective cohort study involving consecutive patients who underwent elective repair of infrarenal AAA between January 2018 and December 2022 at two European vascular centers: the Vascular Surgery Clinic at the University Clinical Center in Gdańsk, Poland, and the VieCuri Medisch Centrum in Venlo, The Netherlands.

**Figure 1:**
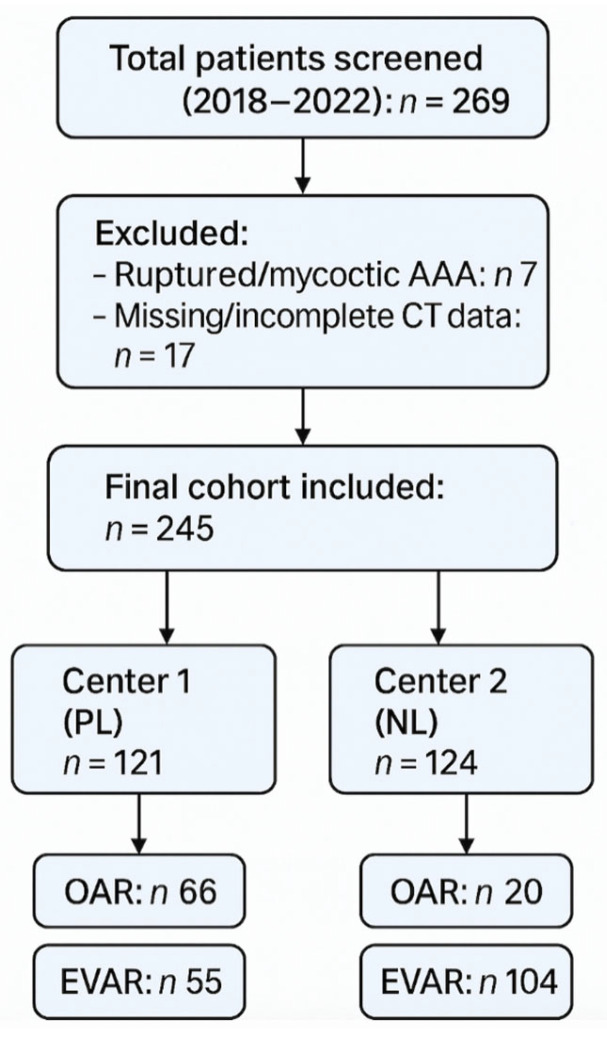
Prisma flowchart.

All patients who underwent either EVAR or OAR during the study period and had available preoperative computed tomography (CT) imaging were eligible for inclusion. Exclusion criteria were ruptured or mycotic aneurysms and incomplete or missing imaging data. A total of 245 patients met the inclusion criteria. Due to the retrospective nature of the study, no formal sample size estimation was performed. Instead, all eligible cases meeting the inclusion criteria from the defined period were included. The study selection process is illustrated in Figure 1. Clinical and demographic data were extracted from electronic medical records, operative reports, and discharge summaries. Collected variables included age, sex, height, body weight, comorbidities (e.g., coronary artery disease, diabetes mellitus, peripheral arterial disease, chronic obstructive pulmonary disease), procedure type, and treatment center. The primary endpoint was the occurrence of early complications during the index hospitalization. These included myocardial infarction, stroke, acute kidney injury, major bleeding, surgical site or systemic infection, unplanned reintervention, and in-hospital death. Long-term outcomes were not evaluated in this study.

### 2.1. Imaging Analysis and Sarcopenia Assessment

Preoperative CT scans were reviewed using OsiriX (Pixmeo, Bernex, Switzerland) and Enterprise Imaging (Agfa HealthCare, Mortsel, Belgium) software (version number: 8.3.2.000). Axial images at the level of the third lumbar vertebra (L3) were used to outline the bilateral borders of the psoas muscles. PMA was calculated in cm^2^. PMI was calculated by dividing the PMA by the square of the patient’s height (in meters), resulting in a value expressed in cm^2^/m^2^. Values were stratified into tertiles separately for each treatment center, as the study populations were found to be too heterogeneous to be analyzed collectively in the primary analysis. Tertile cutoffs were derived from each center’s internal PMI distribution to ensure valid intra-center comparisons. See Supplementary Table S1 for center-specific PMI tertile cutoffs.

### 2.2. Outcomes and Definitions

The primary endpoint was the occurrence of early postoperative complications during the index hospitalization. Complications assessed included myocardial infarction, major bleeding, acute kidney injury, unplanned reintervention, in-hospital death, stroke, infection, wound healing complications, spinal cord ischemia, post-implantation syndrome, type I endoleak, and other early adverse events such as thromboembolic complications, urinary incontinence, bone fractures, and exacerbations of chronic obstructive pulmonary disease. Complications were identified through a retrospective review of inpatient records, operative notes, and discharge summaries. The follow-up period ranged from 1 to 27 days, with a median of 6 days. To ensure consistency and accuracy, all complications were independently verified by two investigators at each participating center. Secondary endpoints included analysis of baseline comorbidity profiles, comparison between open and endovascular repair techniques, and assessment of inter-center differences in complication rates and patient characteristics.

### 2.3. Statistical Analysis

Descriptive statistics were reported as means ± standard deviations for continuous variables and as counts with percentages for categorical variables. Comparisons between groups were performed using Chi-squared tests or Fisher’s exact tests for categorical variables, ANOVA for normally distributed continuous variables, and Mann–Whitney U tests for non-normally distributed continuous variables. To assess the association between PMI tertiles and early complications, analyses were stratified by procedure type and treatment center. To strengthen the findings, we performed a multivariate logistic regression analysis that included variables such as age, sex, comorbidities (hypertension, coronary artery disease, and diabetes), procedure type, center, and PMI tertile. Adjusted odds ratios (aORs) and 95% confidence intervals (CIs) were reported. All statistical analyses were conducted using SAS Studio version 3.81. Statistical significance was defined as a two-tailed *p* < 0.05.

### 2.4. Sample Size and Power

A formal power analysis was not performed due to the retrospective design of the study. Instead, we included all consecutive patients treated between January 2018 and December 2022 at two European vascular centers to maximize external validity and capture the full spectrum of real-world clinical practice.

## 3. Results

A total of 245 patients who underwent AAA repair between January 2018 and December 2022 were included in this study: 121 from the Center 1 and 124 from the Center 2. Center 1 included 30 females (24.8%) and 91 males (75.2%), while Center 2 included 20 females (16.1%) and 104 males (83.9%) (*p* = 0.113). The mean age was significantly lower in Center 1 (70.9 ± 8.7 years) compared to Center 2 (74.3 ± 7.3 years; *p* = 0.006). The prevalence of hypertension was significantly higher in Center 1 (78.5% vs. 50.8%; *p* < 0.0001), while coronary artery disease was more common in Center 2 (41.9% vs. 24.8%; *p* = 0.005). OAR was performed in 66 patients (54.5%) at Center 1 and 20 patients (16.1%) at Center 2, while EVAR was performed in 55 patients (45.5%) at Center 1 and 104 patients (83.9%) at Center 2. Symptomatic presentations were more frequent in Center 1, occurring in 34.8% of OAR patients and 47.3% of EVAR patients, compared to 10.0% (*p* = 0.047) and 6.7% (*p* < 0.0001), respectively, in Center 2. Baseline characteristics of patients treated in Center 1 (Gdańsk) and Center 2 (Venlo) are summarized in Table 1 and Table 2, respectively. A direct comparison of baseline comorbidities and presentation features between the two centers is provided in Table 3.

Early postoperative complications occurred in 27% of patients in Center 1 and 24% in Center 2 (*p* = 0.325). The primary endpoints included myocardial infarction, major bleeding, acute kidney injury, unplanned reintervention, and in-hospital death. Additional complications documented were infection, stroke, wound healing complications, spinal cord ischemia, post-implantation syndrome, type I endoleak, and other events such as thromboembolism, urinary incontinence, fractures, and COPD exacerbations. A detailed breakdown of early postoperative complications, including less frequent events grouped under the ‘Other’ category, is presented in Supplementary Table S3. Supplementary Table S4 summarizes the distribution of early complications stratified by center and procedure type.Complications were identified through a retrospective review of medical records, operative notes, and discharge summaries. Follow-up ranged from 1 to 27 days (median 6 days).

**Figure 2:**
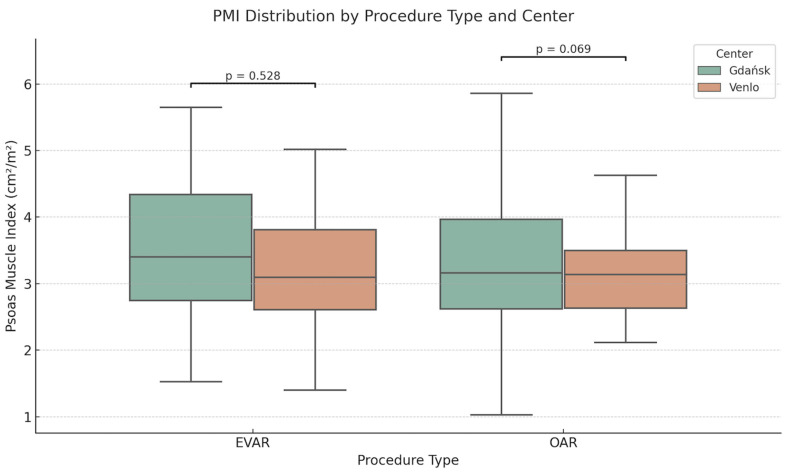
Boxplot showing the distribution of Psoas Muscle Index (PMI) by procedure type and center. PMI was measured from preoperative CT at the L3 level and compared between Gdańsk and Venlo for both EVAR and OAR procedures. *p*-values from Mann–Whitney U tests. Exact numerical values, including group means, standard deviations, and sample sizes, are presented in the Supplementary Materials.

PMI tertiles were defined separately for each center due to population heterogeneity, as described above. The distribution of the PMI by procedure type and center is illustrated in Figure 2. In Center 1, cut-off values were T1 < 2.92 cm^2^/m^2^, T2 2.92–3.71 cm^2^/m^2^, and T3 > 3.71 cm^2^/m^2^; in Center 2, they were T1 < 2.71 cm^2^/m^2^, T2 2.71–3.46 cm^2^/m^2^, and T3 > 3.46 cm^2^/m^2^. The mean PMI was 3.42 ± 0.44 cm^2^/m^2^ in Center 1 and 3.17 ± 0.48 cm^2^/m^2^ in Center 2 (*p* = 0.082). Center-specific PMI tertile cutoffs used for stratification are presented in Supplementary Table S1. Mean PMI values stratified by procedure type and center are reported in Supplementary Table S2.

Complication rates across PMI tertiles varied between procedure types and centers. Among Center 1 OAR patients, early complication rates were 13.0% (T1), 8.7% (T2), and 15.0% (T3) (*p* = 0.808). In Center 2 OAR patients, rates were 42.9% (T1), 42.9% (T2), and 50.0% (T3) (*p* = 0.958). For EVAR patients in Center 1, complication rates were significantly higher in the lowest PMI tertile: 52.9% (T1), 29.4% (T2), and 4.8% (T3) (*p* = 0.004). In contrast, Center 2 EVAR patients showed no significant difference: 17.6% (T1), 17.6% (T2), and 25.0% (T3) (*p* = 0.674).

Multivariate logistic regression confirmed that a low PMI (T1) was independently associated with higher odds of early complications in Center 1 EVAR patients (adjusted OR: 2.88; 95% CI: 1.13–7.34; *p* = 0.026). No significant associations between PMI tertile and complications were found in other subgroups.

## 4. Discussion

Elective AAA repair is a major vascular procedure, typically performed as a prophylactic intervention in elderly, often frail patients with multiple comorbidities [7]. The real challenge in managing AAA lies not in the technical aspects of the procedure, but in selecting appropriate candidates: who is fit for surgery, who might benefit from prehabilitation, and who carries a prohibitive surgical risk [8,9]. Traditional risk models frequently overlook subtle indicators of physiological vulnerability, particularly in high-risk or borderline patients [1,7,10,11]. In clinical practice, a markedly diminished psoas muscle observed on preoperative CT is often interpreted as an intuitive marker of poor physiological reserve. This study examines whether that visual cue, when quantified as a measurable index, holds predictive value in the context of AAA repair. The fact that most AAA procedures are elective offers a useful preoperative period where targeted actions, like nutritional support and structured exercise, could be introduced to enhance outcomes in at-risk patients [12,13].

The PMI has emerged as an indicator of muscle mass and physiological reserve and offers the advantages of rapid assessment, objectivity and easy availability through routine preoperative imaging. Although several studies have emphasized its prognostic potential, the PMI is still far from being a ready-to-use clinical tool. A major limitation is the lack of standardized reference thresholds. Reported thresholds for a “low PMI” vary from 3.24 cm^2^/m^2^ in older cardiovascular patients to over 6.0 cm^2^/m^2^ in younger or healthier individuals. These differences reflect underlying biological differences related to age, gender, ethnicity and general health and raise questions about the generalizability of PMI-based assessments. Ultimately, the diagnostic value of PMI is likely to be context-dependent and determined not only by individual patient characteristics but also by the nature of the procedure and institutional practices.

In our study, a statistically significant association was observed between T1 PMI and early postoperative complications among patients undergoing EVAR at Center 1 (χ^2^ = 11.05, *p* = 0.004). In contrast, no such association was detected for EVAR patients treated at Center 2 (*p* = 0.674), nor for patients undergoing open aneurysm repair (OAR) at either site (Center 1: *p* = 0.808; Center 2: *p* = 0.958). These findings suggest that the PMI value may not be universally applicable. This significant association between low PMI and early complications is observed in only one of the subgroups, illustrating the challenges of applying PMI as a universal prognostic tool. While prior studies have linked low PMI to adverse outcomes after EVAR [11,14], our results caution against overgeneralization. The predictive value of PMI depends on specific clinical contexts, including patient selection, procedural factors, and institutional protocols. Factors such as patient demographics, selection criteria, and institutional care protocols may modify the relationship between muscle mass and surgical risk. The lack of significant associations at Center 2, despite similar methodology, suggests potential influence from unmeasured variables, such as differences in perioperative care, rehabilitation strategies, or simply limited statistical power.

Broader health systems and population-level differences may also play a role. In Poland, where Center 1 is located, the chronic disease burden remains higher than in The Netherlands. For example, smoking rates, obesity prevalence, and physical inactivity are more common in Polish adults compared to their Dutch counterparts [15,16,17]. These public health disparities may increase baseline surgical risk and potentially interact with measures of physiological reserve such as PMI. Interestingly, despite these differences, the average PMI values between centers were not significantly different (PMI: 3.42 vs. 3.17). This suggests that lifestyle and systemic health indicators may influence overall surgical risk more than localized muscle metrics alone. It also underscores the limitations of relying solely on PMI to capture the full complexity of patient vulnerability.

Rather than serving as a stand-alone predictor, PMI may be more effectively utilized as part of a composite risk model that incorporates functional status, comorbidities, and procedural factors. In this context, PMI could contribute to a more personalized preoperative assessment, guiding future strategies such as prehabilitation or nutritional optimization. Further research is needed to determine whether integrating PMI into validated frailty frameworks can improve perioperative outcomes in elderly patients with vascular disease. Future research should incorporate anatomical imaging markers, such as PMI, with validated functional and biochemical measures to enable a more comprehensive and clinically meaningful definition of sarcopenia.

While PMI offers a practical surrogate for muscle mass, it does not capture essential dimensions of muscle function or quality, such as strength or function. Incorporating measures such as handgrip strength, gait speed, or the chair rise test could enhance the prognostic value of imaging data. Additionally, biochemical markers such as serum albumin, creatinine, C-reactive protein, and inflammatory cytokines may provide further insight into nutritional and metabolic status. A multidimensional frailty model that combines PMI with these functional and biochemical indicators could facilitate more precise preoperative risk stratification and help identify patients most likely to benefit from targeted interventions such as prehabilitation or nutritional support. PMI may also serve as a complementary tool to existing frailty assessment instruments. Established scales such as the Fried Frailty Phenotype or the Rockwood Clinical Frailty Scale are widely used to identify at-risk individuals; however, they can be time-consuming, rely on subjective interpretation, and may not always be feasible in acute surgical workflows. In contrast, PMI provides a rapid, objective, and reproducible measure of skeletal muscle mass, which can be readily extracted from preoperative CT scans routinely performed in vascular surgery. When used in conjunction with conventional frailty tools, PMI may enhance the accuracy and efficiency of preoperative risk stratification. Future studies should explore how integrating anatomical and functional metrics of frailty improves the prediction of surgical outcomes. The discrepancy in findings between centers underscores the context-dependent nature of PMI as a risk marker. Differences in perioperative protocols, patient selection, and underlying health status likely contributed to the observed variation. Rather than invalidating PMI as a biomarker, these results emphasize the need for institution-specific validation and cautious interpretation when applying sarcopenia metrics across diverse clinical settings.

This study is an early contribution toward clarifying the diagnostic value of PMI in vascular surgery. Its lack of universal cutoffs is not a flaw but a reflection of patient diversity. Rather than expecting PMI to work the same in every context, future research should define where and how it adds the most clinical value [1,2,18,19,20,21]. The association between lower PMI and early postoperative complications was observed only in EVAR patients at one center, underscoring the context-dependent nature of this metric. Our results support the need for validation in larger, prospective, multicenter studies using standardized definitions of sarcopenia and harmonized perioperative protocols. As a retrospective, real-world analysis, this study does not establish definitive thresholds but instead delineates the conditions under which PMI may provide prognostic insight. Such nuance is essential for the development of personalized, imaging-informed risk models in vascular surgery.

### Limitations

No initial power calculation was conducted because this was a retrospective observational study of consecutive patients treated at two vascular centers. While our total sample size is comparable to similar studies on sarcopenia in vascular surgery, which generally include 100–300 patients [2,4], the risk of Type II error persists, particularly in groups with smaller sample sizes. We acknowledge this limitation when interpreting our results.

Unmeasured variables, such as frailty scores, functional capacity, inflammatory markers, or socioeconomic factors, may have influenced the observed outcomes but were not available for inclusion in our model.

Although PMI was calculated using a standardized and reproducible technique from routine preoperative CT scans, the absence of universally accepted thresholds for “low” PMI limits its clinical interpretability. To address population heterogeneity, we stratified PMI into tertiles within each center, but these relative categories may not correspond to validated diagnostic criteria for sarcopenia or low muscle mass. As such, our findings cannot be used to propose specific cut-off values or directly inform patient management without further external validation.

Finally, our analysis was limited to anatomical imaging-derived measures of sarcopenia. Functional or biochemical markers, such as grip strength, gait speed, albumin levels, or inflammatory markers, were unavailable for this retrospective study. As such, we were unable to integrate PMI into a broader diagnostic model of frailty or to correlate muscle mass with strength and performance. Future prospective studies should incorporate multidimensional frailty assessments and evaluate whether combining anatomical, functional, and molecular markers improves perioperative risk prediction.

## 5. Conclusions

PMI may serve as a useful imaging-based biomarker of physiological reserve in selected patients undergoing EVAR, but its predictive value is highly context-dependent. Rather than being used in isolation, PMI should be integrated into comprehensive risk models and validated in multicenter, prospective studies before clinical adoption.

## Figures and Tables

**Table 1:** Patient characteristics in Center 1.

Variable	Value	%
Number of Patients	121	
Age (mean ± SD)	70.9 ± 8.7	
Male	91	75%
Female	30	25%
OAR	66	54.5%
EVAR	55	45.5%

**Table 2:** Patient characteristics in Center 2.

Variable	Value	%
Number of Patients	124	
Age (mean ± SD)	74.32 ± 7.3	
Male	104	84%
Female	20	16%
OAR	20	16.1%
EVAR	104	83.9%

**Table 3:** Comparison of comorbidities and presentation characteristics between patients treated at Center 1 (Gdańsk, Poland) and Center 2 (Venlo, The Netherlands).

Comorbidity	Center 1 (%)	Center 2 (%)	*p*-Value
Hypertension	78.5	50.8	<0.0001
Coronary artery disease	24.8	41.9	<0.005
Myocardial infarction	13.2	21.0	0.128
Previous coronary revascularization	16.5	29.0	<0.023
Peripheral artery disease	7.4	21.8	<0.002
Chronic heart failure	8.3	8.9	1.000
Hyperlipidemia	15.7	32.3	0.003
Chronic obstructive pulmonary disease	9.1	19.4	0.028
Chronic kidney disease	10.7	8.9	0.670
Current or former smoker	55.4	48.4	0.307
Symptomatic presentation	40.5	15.5	<0.0001

## Data Availability

Data are available on request from the author.

## Supplementary Materials

The following supporting information can be downloaded at https://www.mdpi.com/article/10.3390/jcm14155333/s1, Supplementary Table S1. Center-specific PMI tertile cutoffs; Supplementary Table S2. Mean PMI values by procedure type and center; Supplementary Table S3. Detailed breakdown of early postoperative complications. Values represent absolute counts and percentages relative to the total study population. Multiple complications per patient were possible. The “Other” category includes early thromboembolic events, urinary incontinence, bone fractures occurring in the early postoperative period, and exacerbations of chronic obstructive pulmonary disease (COPD) occurring in the early postoperative period; Supplementary Table S4. Early complications by center and procedure type.

## Abbreviations

The following abbreviations are used in this manuscript:

Abbreviation	Full Term
AAA	Abdominal Aortic Aneurysm
AGFA	AGFA Enterprise Imaging
CAD	Coronary Artery Disease
CI	Confidence Interval
CT	Computed Tomography
EVAR	Endovascular Aneurysm Repair
L3	Third Lumbar Vertebra
OAR	Open Aneurysm Repair
OECD	Organisation for Economic Co-operation and Development
PAD	Peripheral Artery Disease
PMA	Psoas Muscle Area
PMD	Psoas Muscle Density
PMI	Psoas Muscle Index
SD	Standard Deviation
TIA	Transient Ischemic Attack
WHO	World Health Organization
aOR	Adjusted Odds Ratio
