# Supplementary material for: Computed Tomography-Derived Psoas Muscle Index as a Diagnostic Predictor of Early Complications Following Endovascular Aortic Repair: A Retrospective Cohort Study from Two European Centers
Source: J Clin Med. 2025 Jul 28;14(15):5333. doi: 10.3390/jcm14155333 (PMC12346985; doi:10.3390/jcm14155333)
Supplement: Supplementary file 1 [file jcm-14-05333-s001.zip › jcm-3682561-supplementary.pdf]

Journal of
Clinical Medicine

Supplementary material:

Supplementary Table S1. Center-Specific PMI Tertile Cutoffs

Center	T1 (PMI <)	T2 (PMI 33–66%)	T3 (PMI >)
Gdańsk (Center 1)	2.92 cm²/m²	2.92–3.71 cm²/m²	> 3.71 cm²/m²
Venlo (Center 2)	2.71 cm²/m²	2.71–3.46 cm²/m²	> 3.46 cm²/m²

Supplementary Table S2. Mean PMI Values by Procedure Type and Center

Procedure	Center	Mean PMI (cm²/m²)	SD	n
EVAR	Gdańsk (Center 1)	3.5	1.02	56
EVAR	Venlo (Center 2)	3.17	0.82	104
OAR	Gdańsk (Center 1)	3.32	0.99	67
OAR	Venlo (Center 2)	3.14	0.75	20

Supplementary Table S3. Detailed breakdown of early postoperative complications. Values represent absolute counts and percentages relative to the total study population. Multiple complications per patient were possible. The Other" category includes early thromboembolic events, urinary incontinence, bone fractures occurring in the early postoperative period, and exacerbations of chronic obstructive pulmonary disease (COPD) occurring in the early postoperative period.

Complication	Count	Percentage (%)
Myocardial Infarction	0	0.0
Stroke	3	4.6
Bleeding	6	9.2
Acute Kidney Injury (AKI)	4	6.2

Reintervention	5	7.7
Infection	16	24.6
Post-implantation Syndrome	2	3.1
Wound Healing Complications	4	6.2
Spinal Cord Ischemia (SCI)	3	4.6
Death	5	7.7
Endoleak Type I	3	4.6
Other	14	21.5
total	65	100

Supplementary Table S4. Early Complications by Center and Procedure Type

Procedure Type	Center	Total Procedures	Complicated Cases (n)	Complication Rate (%)
Open Repair	1	66	20	30.3%
Open Repair	2	20	9	45.0%
EVAR	1	55	15	27.3%
EVAR	2	104	21	20.2%
